# Risk factors for type 1 and type 2 myocardial infarction

**DOI:** 10.1093/eurheartj/ehab581

**Published:** 2021-08-25

**Authors:** Ryan Wereski, Dorien M Kimenai, Anda Bularga, Caelan Taggart, David J Lowe, Nicholas L Mills, Andrew R Chapman

**Affiliations:** BHF Centre for Cardiovascular Science, University of Edinburgh, Chancellors Building, 49 Little France Crescent, Edinburgh EH16 4SA, UK; Usher Institute, University of Edinburgh, Edinburgh, NINE, 9 Little France Road, Edinburgh BioQuarter, Edinburgh EH16 4UX, UK; BHF Centre for Cardiovascular Science, University of Edinburgh, Chancellors Building, 49 Little France Crescent, Edinburgh EH16 4SA, UK; BHF Centre for Cardiovascular Science, University of Edinburgh, Chancellors Building, 49 Little France Crescent, Edinburgh EH16 4SA, UK; University of Glasgow, School of Medicine, Glasgow, UK; BHF Centre for Cardiovascular Science, University of Edinburgh, Chancellors Building, 49 Little France Crescent, Edinburgh EH16 4SA, UK; Usher Institute, University of Edinburgh, Edinburgh, NINE, 9 Little France Road, Edinburgh BioQuarter, Edinburgh EH16 4UX, UK; BHF Centre for Cardiovascular Science, University of Edinburgh, Chancellors Building, 49 Little France Crescent, Edinburgh EH16 4SA, UK

**Keywords:** Myocardial infarction, Risk factors, Universal definition, Acute coronary syndrome, Type 2

## Abstract

**Aims:**

Whilst the risk factors for type 1 myocardial infarction due to atherosclerotic plaque rupture and thrombosis are established, our understanding of the factors that predispose to type 2 myocardial infarction during acute illness is still emerging. Our aim was to evaluate and compare the risk factors for type 1 and type 2 myocardial infarction.

**Methods and results:**

We conducted a secondary analysis of a multi-centre randomized trial population of 48 282 consecutive patients attending hospital with suspected acute coronary syndrome. The diagnosis of myocardial infarction during the index presentation and all subsequent reattendances was adjudicated according to the Universal Definition of Myocardial Infarction. Cox regression was used to identify predictors of future type 1 and type 2 myocardial infarction during a 1-year follow-up period. Within 1 year, 1331 patients had a subsequent myocardial infarction, with 924 and 407 adjudicated as type 1 and type 2 myocardial infarction, respectively. Risk factors for type 1 and type 2 myocardial infarction were similar, with age, hyperlipidaemia, diabetes, abnormal renal function, and known coronary disease predictors for both (*P* < 0.05 for all). Whilst women accounted for a greater proportion of patients with type 2 as compared to type 1 myocardial infarction, after adjustment for other risk factors, sex was not a predictor of type 2 myocardial events [adjusted hazard ratio (aHR) 0.82, 95% confidence interval (CI) 0.66–1.01]. The strongest predictor of type 2 myocardial infarction was a prior history of type 2 events (aHR 6.18, 95% CI 4.70–8.12).

**Conclusions:**

Risk factors for coronary disease that are associated with type 1 myocardial infarction are also important predictors of type 2 events during acute illness. Treatment of these risk factors may reduce future risk of both type 1 and type 2 myocardial infarction.

## Graphical Abstract

**Graphical Abstract:**
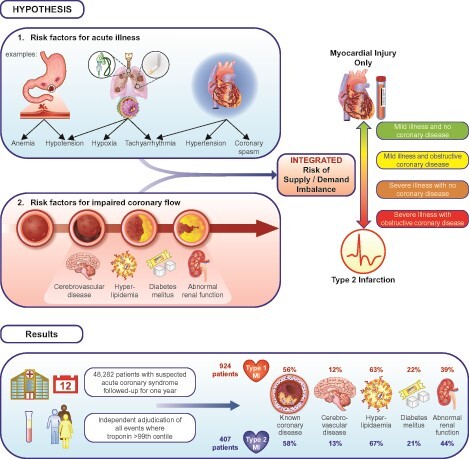
Determining vulnerability to future type 2 myocardial infarction events. The role of illness severity, risk factors for cardiovascular disease, and presence of coronary artery disease in those with type 2 myocardial infarction.

See the editorial comment for this article ‘Risk Factors for Type 2 MI: The Usual Suspects or Guilt by Association?’, by Michelle L. O’Donoghue et al., https://doi.org/10:1093/eurheartj/ehab707.

## Introduction

Myocardial infarction is a clinical diagnosis based on the presence of symptoms or signs of myocardial ischaemia in conjunction with acute myocardial injury, as indicated by a rise or fall in cardiac biomarker concentrations.^1^ The Fourth Universal Definition recognizes that myocardial infarction can result from a number of different pathophysiological mechanisms.^1–3^ Type 1 myocardial infarction occurs in those with atherosclerotic plaque rupture and thrombosis, whereas type 2 myocardial infarction occurs due to myocardial oxygen supply and demand imbalance in the context of an acute illness causing tachyarrhythmia, hypoxia, or hypotension without acute atherothrombosis.

The risk factors for type 1 myocardial infarction have been well characterized in a number of large trials.^4^ ^,^ ^5^ Pre-existing conditions such as hypertension, diabetes mellitus, and hyperlipidaemia have been shown to increase future atherothrombotic risk. Risk factors that predict type 2 myocardial infarction are likely to be more complex, with an acute illness responsible for supply or demand imbalance and myocardial infarction in a population of susceptible patients. Supply or demand imbalance itself is often multifactorial and may arise from any acute process leading to sustained tachycardia, hypoxia, or hypotension. However, type 2 myocardial infarction is not ubiquitous during acute illness, and some patients may tolerate significant haemodynamic stress without cardiac consequence (*Graphical Abstract*).

Our knowledge of the risk factors and patient characteristics that predispose to type 2 myocardial infarction during acute illness is limited.^6–11^ Meanwhile, clinical outcomes after type 2 myocardial infarction remain poor, with as many as two-third of patients dying within 5 years.^7^ ^,^ ^9^ ^,^ ^12–14^ A greater understanding of risk factors for type 2 myocardial infarction could facilitate targeted intervention and perhaps reduce the long-term risk of future events in this population. Here, we evaluate the predictors of future type 1 and type 2 myocardial infarction events during 1-year follow-up of a population of consecutive patients who attended hospital with suspected acute coronary syndrome.

## Methods

### Study population

Between June 2013 and March 2016, a total of 48 282 consecutive patients with suspected acute coronary syndrome who attended one of the 10 secondary and tertiary care hospitals in Scotland were enrolled in the High-Sensitivity Troponin in the Evaluation of patients with Acute Coronary Syndrome (High-STEACS) trial.^15^ All patients attending the emergency department were screened for suspected acute coronary syndrome by the attending clinician. To avoid selection bias and ensure a representative population we used an enrolment tool embedded into the electronic health care system. Patients were eligible for inclusion if they presented with suspected acute coronary syndrome and had cardiac troponin measured using a high-sensitivity cardiac troponin I assay (hs-cTnI; ARCHITECT_*STAT*_ high-sensitive troponin I assay; Abbott Park, IL, USA). This assay has an inter-assay coefficient of variation of <10% at 4.7 ng/L, the limit of detection of 1.2 and 1.9 ng/L, and a sex-specific 99th centile thresholds of 16 and 34 ng/L for women and men, respectively.^16^ ^,^ ^17^ Standardized practice guidelines were in place across all recruiting centres during study enrolment. In accordance with guidelines, myocardial infarction was ruled out when cardiac troponin concentrations were <99th percentile at presentation if symptom onset was >6 h from presentation or after serial testing 6–12 h from symptom onset in those presenting earlier.^18^ ^,^ ^19^ Patients were excluded if they were not resident in Scotland or had been enrolled in the trial previously. Following discharge, all patients who reattended with suspected acute coronary syndrome were identified using the same electronic screening tool.^15^ We used regional and national registries to ensure complete follow-up for the study population over a 1-year period from the date of the index admission.

### Adjudication of myocardial infarction

All diagnoses in patients with hs-cTnI concentrations above the sex-specific 99th centile were adjudicated using the hs-cTnI assay and classified according to the Fourth Universal Definition of Myocardial Infarction.^1^ Two physicians independently reviewed all clinical information, blinded to study phase, with discordant diagnoses resolved by a third reviewer. Type 1 myocardial infarction was defined as myocardial necrosis (any hs-cTnI concentration above the sex-specific 99th centile with a rise or fall in hs-cTnI concentration where serial testing was performed) in the context of a presentation with suspected acute coronary syndrome and either symptoms of myocardial ischaemia or signs on the electrocardiogram. Patients with myocardial necrosis, symptoms, or signs of myocardial ischaemia and evidence of increased myocardial oxygen demand or decreased supply secondary to an alternative condition without evidence of acute atherothrombosis were defined as type 2 myocardial infarction. Patients with hs-cTnI concentrations above the 99th centile without symptoms or signs of myocardial ischaemia were classified as having myocardial injury. Here, the final clinical diagnosis was also adjudicated according to prespecified criteria. All non-ischaemic myocardial injury was classified as acute, unless a change of ≤20% was observed on serial testing, or the final adjudicated diagnosis was chronic heart failure or chronic renal failure, where the classification was chronic myocardial injury. Abnormal renal function was defined in patients with an estimated glomerular filtration rate <60 mL/min/1.73 m^2^. A detailed summary of the adjudication procedures is provided in the Supplementary material online, *Appendix*.

### Statistical analysis

Baseline characteristics were summarized for the whole population, and in those with and without myocardial infarction during follow-up. Continuous variables are presented as mean (standard deviation) or median [interquartile range (IQR)], as appropriate. Categorical variables are presented as absolute numbers (%). Group-wise comparisons were performed using *χ* ^2^, Kruskal–Wallis, or one-way analysis of variance tests as appropriate. Multivariable Cox proportional hazard models were used to determine the predictors of type 1 or type 2 myocardial infarction accounting for the competing risk of all-cause mortality. Adjusted hazard ratios (aHRs) are reported with 95% confidence intervals (CIs) and *P*-values for the individual outcomes of type 1 or type 2 myocardial infarction. Baseline clinical variables for the models were selected based on their clinical relevance and included age, sex, previous history of coronary artery disease, previous history of cerebrovascular disease, diabetes mellitus, hyperlipidaemia, creatinine concentration, and coronary revascularization history.^6^ ^,^ ^10^ ^,^ ^12^ ^,^ ^20^ ^,^ ^21^ We also included an additional term for the diagnosis at index admission and calculated the hazard of subsequent type 1 or type 2 for each index diagnosis vs. patients with no myocardial injury. Age and creatinine concentration were modelled as continuous variables. Data were assessed for systematic missingness, and complete case analysis was used in Cox regression models with no data imputation. We examined for non-proportional hazards graphically and by calculating deviation from linearity over time using Schoenfeld residuals, where individual covariate *P*-values >0.05 were considered satisfactory to meet the proportional hazards assumption. Statistical analysis was performed in R (version 3.5.1).

### Approvals and ethics

This study was conducted in accordance with the Declaration of Helsinki and approved by the Scotland A Research Ethical Committee. All data were collected prospectively from the electronic patient records, deidentified, and linked within secure NHS Safe Havens (*Supplementary material online, Table S4*).

## Results

In 48 282 patients with 1 year of follow-up, 2839 patients reattended the emergency department with suspected acute coronary syndrome and were found to have a hs-cTnI concentration above the 99th centile. It was possible to adjudicate the index and subsequent diagnosis in 88% and 92% of patients, respectively, with moderate agreement between adjudicators for the index (Cohen’s kappa coefficient, *K* = 0.8) and subsequent diagnoses (*K* = 0.7). During follow-up, there were 924 patients with an adjudicated diagnosis of type 1 myocardial infarction, 407 with type 2 myocardial infarction, and 1169 and 77 patients adjudicated as acute or chronic myocardial injury, respectively.

Patients with either type 1 or type 2 myocardial infarction had a similar prevalence of known coronary disease (56% vs. 58%), cerebrovascular disease (12% vs. 13%), hyperlipidaemia (63% vs. 67%), and diabetes mellitus (22% vs. 21%) (*Table 1*). However, compared to those with type 1 myocardial infarction, those with type 2 myocardial infarction were marginally older [median age 77 (IQR 69–83) vs. 74 (IQR 62–83)] and were more likely to have abnormal renal function (44% vs. 39%).

**Table 1:** Characteristics of patients with and without myocardial infarction during follow-up

	All patients	No myocardial infarction	Type 1 myocardial infarction	Type 2 myocardial infarction
Number of participants	48 282 (100)	46 929 (97)	924 (2)	407 (1)
Age (years), median (IQR)	61 (49–65)	61 (17)	74 (62–83)	77 (69–83)
Male sex	25 720 (53)	24 996(53)	521 (56)	189 (46)
Medical history prior to index presentation				
Coronary artery disease	11 912 (25)	11 147 (24)	521 (56)	236 (58)
Cerebrovascular disease	2949 (6)	2783 (6)	112 (12)	53 (13)
Hyperlipidaemia	19 366 (40)	18 502 (39)	581 (63)	273 (67)
Diabetes mellitus	3518 (7)	3229 (7)	201 (22)	85 (21)
Abnormal renal function	9325 (20)	8798 (19)	348 (39)	175 (44)
Creatinine concentration (μmol/L), median (IQR)	75 (66–91)	73 (65–86)	83 (71–105)	92 (72–122)
PCI	3682 (8)	3451 (7)	164 (18)	63 (16)
CABG	782 (2)	732 (2)	39 (4)	11 (3)
Prior myocardial infarction or injury				
Type 1 MI	4981 (11)	4477 (10)	409 (46)	83 (21)
Type 2 MI	1121 (2)	1007 (2)	39 (4)	75 (19)
Acute myocardial injury	1676 (4)	1592 (4)	46 (5)	38 (10)
Chronic myocardial injury	1287 (3)	1219 (3)	43 (5)	25 (7)
No myocardial injury	37 922 (79)	37 404 (82)	344 (39)	166 (43)
Medical therapies				
ACE inhibitor/ARB	18 329 (38)	17 453 (37)	607 (66)	254 (62)
Beta blocker	17 589 (36)	16 717 (36)	599 (65)	262 (64)
Aspirin	17 124 (36)	16 189 (35)	654 (71)	265 (65)
P2Y_12_ inhibitor	8705 (18)	8087 (17)	478 (52)	135 (32)
Oral anticoagulant	4730 (10)	4502 (10)	124 (13)	103 (25)
Spironolactone	1612 (3)	1496 (3)	74 (8)	42 (10)
Lipid-lowering therapy	22 427 (47)	21 406 (46)	706 (76)	297 (73)

Values are shown as *n* (%) unless otherwise stated.

ACE, angiotensin-converting enzyme; ARB, angiotensin receptor blocker; CABG, coronary artery bypass graft; IQR, interquartile range; MI, myocardial infarction; PCI, percutaneous coronary intervention.

It was possible to determine the cause of subsequent type 2 myocardial infarction in 98% (397/407) of patients. Tachyarrhythmia (50%), hypoxia (22%), hypotension (12%), and anaemia (11%) were the most common causes, with coronary causes (<2%) and hypertension (2%) less frequent (Supplementary material online, *Figure S1* and *Table S1*). There was no difference in age, sex, prior history of coronary disease, cerebrovascular disease, hyperlipidaemia, or diabetes mellitus between different causes of type 2 myocardial infarction (Supplementary material online, *Table S2*).

**Figure 1:**
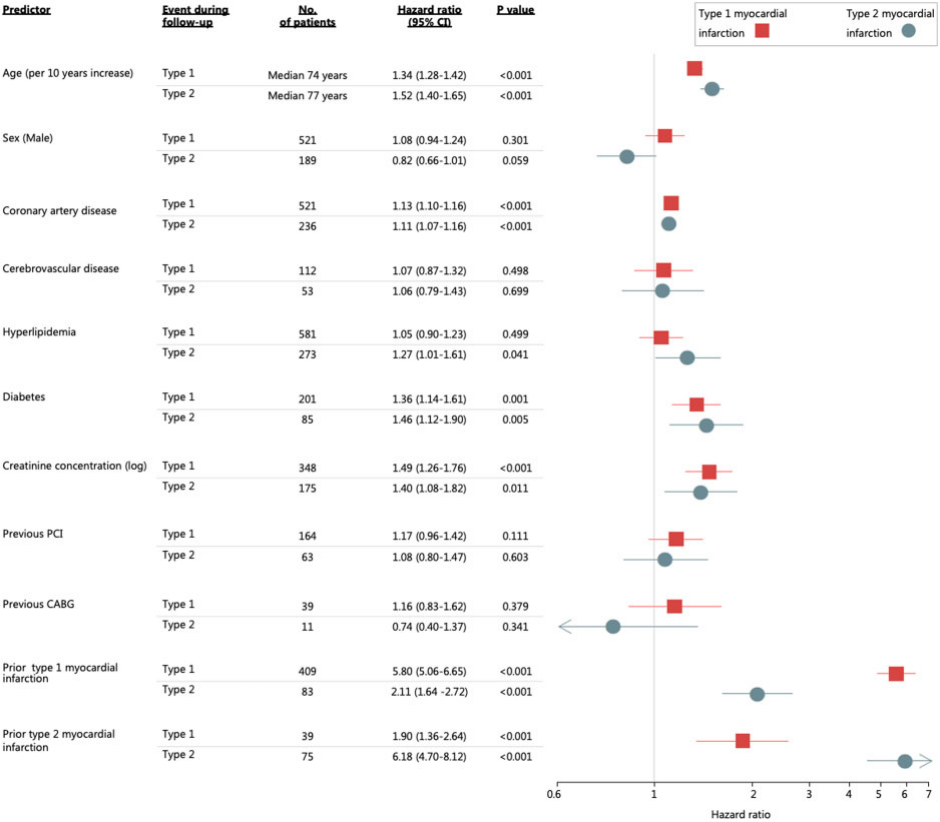
Predictors of future type 1 myocardial infarction and type 2 myocardial infarction. Age is per 10 years, modelled as a continuous variable. CI, confidence interval; CABG, coronary artery bypass graft; PCI, percutaneous coronary intervention.

In a multivariable model, the factors associated with increased susceptibility for future type 1 or type 2 myocardial infarction were similar (*Figure 1*). Risk factors for type 1 myocardial infarction were increased age (aHR 1.34, 95% CI 1.28–1.42) and a prior history of coronary artery disease (aHR 1.13, 95% CI 1.10–1.16), diabetes mellitus (aHR 1.36, 95% CI 1.14–1.61), or increased creatinine concentration (aHR 1.49, 95% 1.26–1.76 per log increase) (*Figure 1*). Similarly, type 2 myocardial infarction was more likely in those with increased age (aHR 1.52, 95% CI 1.40–1.65), or a prior history of coronary artery disease (aHR 1.11, 95% CI 1.07–1.16), hyperlipidaemia (aHR 1.27, 95% CI 1.01–1.61), diabetes mellitus (aHR 1.46, 95% CI 1.12–1.90), or increased creatinine concentration (aHR 1.40, 95% CI 1.08–1.82 per log increase). Sex was not a significant predictor of type 1 (aHR 1.08, 95% CI 0.94–1.24) or type 2 (aHR 0.82, 95% CI 0.66–1.01) myocardial infarction.

**Figure 2:**
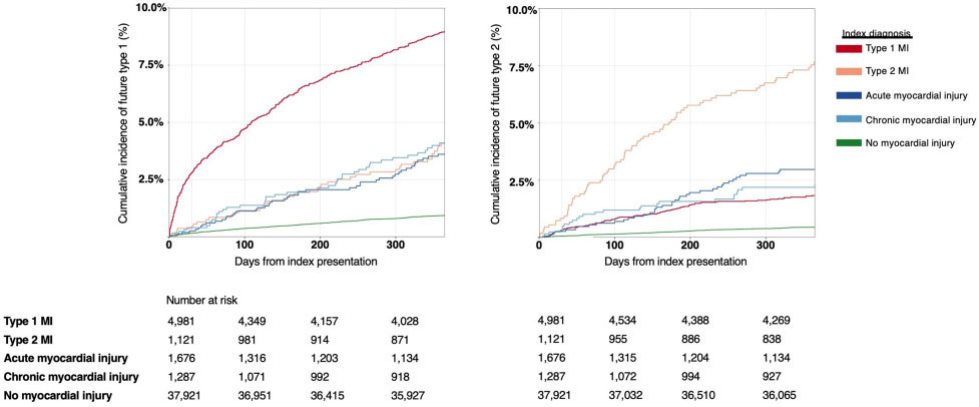
Cumulative incidence curves illustrating the risk of future type 1 (left panel) and future type 2 myocardial infarction (right panel), stratified by prior diagnosis, with number at risk of event in corresponding tables. MI, myocardial infarction.

A prior diagnosis of type 1 or type 2 myocardial infarction was the strongest predictor of future type 1 or type 2 events, respectively. Patients with a prior type 1 myocardial infarction had the highest risk of a future type 1 myocardial infarction event (aHR 5.80, 95% CI 5.06–6.65) with a subsequent type 1 event occurring in 8% (409/4981) of those with a prior type 1 myocardial infarction, as compared to 3% (39/1121) of those with a prior type 2 event (*Figure 2*). Similarly, patients with a prior type 2 myocardial infarction were at increased risk of a future type 2 event (aHR 6.18, 95% CI 4.70–8.12). Future type 2 events occurred in 7% (75/1121) of patients with a prior type 2 event, as compared to 2% (83/4981) of those with a prior type 1 myocardial infarction (*Figure 2*).

Patients with a future type 1 or 2 myocardial infarction during follow-up were on similar preventative medication including aspirin (71% vs. 65%), lipid-lowering therapy (76% vs. 73%), beta-blockers (65% vs. 64%), and angiotensin-converting enzyme inhibitors (66% vs. 62%, *Table 1*).

Further details of patients with recurrent myocardial infarction can be found in the *Supplementary material online, Table S3*.

## Discussion

In 48 282 patients with suspected acute coronary syndrome, we determined the risk factors associated with development of future type 1 or type 2 myocardial infarction. We report several observations that are informative for clinical practice. We observed that risk factors traditionally associated with type 1 myocardial infarction, including diabetes mellitus, hyperlipidaemia, abnormal renal function, and a prior history of coronary artery disease, were also important predictors of type 2 myocardial infarction. Whilst all patients with a prior history of myocardial infarction were at increased risk of recurrent events, patients with a previous type 2 myocardial infarction were six times more likely to have a subsequent type 2 event. The similar cardiovascular risk factor profile of patients with future type 1 or type 2 myocardial infarction suggests that shared underlying pathophysiological mechanisms may increase the susceptibility for type 2 myocardial infarction. Identifying and targeting these risk factors in patients with type 2 myocardial infarction, for example, through optimizing glycaemic control, blood pressure, lipid levels, and ensuring those with underlying coronary disease are on optimal secondary prevention, could reduce the risk of future events.

The risk factors for type 1 myocardial infarction were established in a number of large studies predating the introduction of high-sensitivity cardiac troponin and the Universal Definition of Myocardial Infarction.^4^ ^,^ ^5^ In these studies, enrolment was either selective or restricted to patients in coronary care units, and those with significant comorbidities were excluded. This resulted in preferential recruitment of those with atherothrombotic type 1 myocardial infarction. Pre-existing conditions such as hypertension, diabetes mellitus, and hyperlipidaemia increase the risk of type 1 myocardial infarction, and the modification of these risk factors through lifestyle intervention and pharmacotherapy has been shown to reduce future type 1 myocardial infarction events and improve survival.^5^ ^,^ ^22^

In contrast, knowledge of the factors that predispose to type 2 myocardial infarction is still emerging. A number of cross-sectional studies have compared the characteristics of patients classified with either type 1 or type 2 myocardial infarction,^6–11^ ^,^ ^23^ but to date there have been no prospective studies evaluating the risk factors for future type 1 or type 2 myocardial infarction events during follow-up. Unlike type 1 myocardial infarction, which is caused by atherothrombotic plaque rupture, type 2 myocardial infarction has a more diverse and complex aetiology. Our findings are consistent with those of Saaby *et al.* ^24^ who demonstrated that patients with type 2 myocardial infarction most commonly presented with tachyarrhythmia (29%), anaemia (21%), hypoxia (21%), or hypotension (6%).

Although the underlying conditions responsible for myocardial oxygen supply or demand imbalance are heterogeneous, our analysis is the first to demonstrate that a number of common cardiovascular risk factors are associated with susceptibility to type 2 myocardial infarction. We demonstrate that several risk factors usually associated with coronary disease and atherothrombotic type 1 myocardial infarction, including hyperlipidaemia, diabetes mellitus, and a prior history of coronary artery disease, are also important predictors of future type 2 myocardial infarction (*Figure 1*). Our findings are consistent with prior studies of consecutive patients with myocardial infarction where investigators demonstrated that >50% of patients undergoing coronary angiography had evidence of obstructive coronary disease.^24^ ^,^ ^25^ Whilst a number of studies have reported that coronary artery disease and its risk factors are common in patients presenting with type 2 myocardial infarction,^26^ ^,^ ^27^ only one previous study has prospectively evaluated the link with future type 2 events. CASABLANCA is a prospective cohort study that enrolled patients who underwent peripheral or coronary angiography and were followed up for incident myocardial infarction events. This demonstrated that patients with future type 2 myocardial infarction were more likely to have multivessel obstructive disease at baseline, compared to those without a future type 2 myocardial infarction event (47.7% vs. 35.3%).^12^

Our observation that traditional cardiovascular risk factors were common and shared predictors of future type 1 or type 2 myocardial infarction suggests that the presence of coronary artery disease, or risk factors for the progression of atherosclerosis, confers increased susceptibility to type 2 events. Whilst the risk of supply or demand imbalance leading to type 2 myocardial infarction is primarily driven by the severity of acute physiological stress, the threshold at which ischaemia occurs may be related to the extent of flow limitation from atherosclerotic coronary disease. In contrast to a patient with obstructive coronary artery disease and attenuated flow reserve, a patient with no coronary disease may have a higher ischaemic threshold, and a similar physiological stress may not lead to cardiac consequences.^28^ This underlines the importance of considering the magnitude of supply or demand imbalance and probability of coronary artery disease relative to each individual patient (*Graphical Abstract*). Furthermore, coronary artery disease in those with type 2 myocardial infarction could explain why the incidence of future cardiac death is broadly comparable to patients with type 1 events.^7^ ^,^ ^10^ This raises the possibility that therapies and interventions to reduce the burden of coronary disease, including coronary revascularization, could modify the risk of myocardial ischaemia and play an important role in reducing the risk of type 2 myocardial infarction and adverse outcomes.

Currently, there is no consensus on how patients with type 2 myocardial infarction should be managed. In contrast to type 1 myocardial infarction where ∼60% patients undergo coronary angiography and 80% are prescribed preventative pharmacotherapies at discharge, in those with type 2 myocardial infarction, <20% of patients undergo coronary angiography and >40% are discharged without antiplatelet or lipid-lowering therapies.^23^ ^,^ ^29^ In the present study, we observed that the majority of patients hospitalized for type 2 myocardial infarction were already established on cardioprotective pharmacotherapies. This is not overly surprising given a previous myocardial infarction was the most important predictor of future events during follow-up. It is likely that the proportion of patients with type 2 myocardial infarction already established on preventative therapies would be much lower in the general population as has been observed in prior studies.^8^ ^,^ ^9^ ^,^ ^29^

Whether the systematic use of preventative therapies or treatments for coronary artery disease, or its risk factors, can reduce the risk of type 2 myocardial infarction is uncertain. Indeed, widespread use of antiplatelet medication could be harmful in type 2 myocardial infarction, including, for example, where acute supply or demand imbalance has been triggered by bleeding.^30^ Careful selection of patients with type 2 myocardial infarction for any therapies should be undertaken after recovery from acute illness. The use of registry and cohort studies to evaluate this question is inherently challenging with significant confounding by indication, and dedicated randomized controlled trials of drug therapies in selected patients with type 2 myocardial infarction are needed.

Despite the paucity of trials in type 2 myocardial infarction, exploratory analyses from trials in other populations have shown that the treatment of risk factors for coronary artery disease may reduce the risk of type 2 myocardial infarction. The ODYSSEY OUTCOMES trial recently observed that alirocumab, a proprotein convertase subtilisin/kexin type 9 inhibitor used in the treatment of hypercholesterolaemia, could reduce both future type 1 and type 2 myocardial infarction by 13% and 22%, respectively, as compared to placebo.^31^ Furthermore, in the DECLARE-TIMI 58 trial, dapagliflozin, an sodium-glucose co-transporter 2 inhibitor, reduced future type 1 and type 2 myocardial infarction in patients with a history of myocardial infarction and type 2 diabetes mellitus.^32^ These observations suggest that interventions that reduce cardiovascular risk through multiple mechanisms, including reducing the burden of hypercholesterolaemia or optimizing management of diabetes, should be evaluated for their effectiveness in dedicated trials of carefully selected patients with type 2 myocardial infarction.

Our observation that type 2 myocardial infarction begets type 2 myocardial infarction could be a consequence of the lower rates of coronary angiography, revascularization, and pharmacotherapy prescriptions in these patients.^29^ Failure to identify and treat important coronary disease in patients with type 2 myocardial infarction could result in progressive worsening of ischaemic burden and increased risk of recurrent infarction during future acute illness. It is therefore plausible that wider use of both invasive and non-invasive coronary imaging could facilitate improved identification of coronary disease and help target treatments designed to improve outcomes and reduce the risk of future events in patients with type 2 myocardial infarction. The Appropriateness of Coronary Investigation in Myocardial Injury and Type 2 Myocardial Infarction (ACT-2) randomized controlled trial will assess whether computed tomography or invasive coronary angiography might improve the prescription of secondary prevention therapy and impact on future cardiovascular outcomes.^33^

In our study, we also evaluated whether sex was associated with the risk of myocardial infarction. Male sex is an established risk factor for type 1 myocardial infarction,^34^ but the role of sex in type 2 myocardial infarction is unclear. Consistent with previous studies, we observed that, when compared to type 1 myocardial infarction, women account for a higher proportion of patients with type 2 events.^6–11^ ^,^ ^24^ ^,^ ^35^ ^,^ ^36^ However, when adjusting for cardiovascular risk factors and comorbidities, sex was not an important predictor of type 2 myocardial infarction. This observation may not be entirely surprising given the acute triggers for type 2 events, such as tachycardia, hypoxia, and hypotension, are perhaps less likely to be influenced by patient sex. Although this observation contrasts the findings of Neumann *et al.* ^11^ who reported in a cross-sectional analysis that female sex was associated with type 2 myocardial infarction, data from the CASABLANCA study, which is the only other prospective evaluation of risk factors for type 2 myocardial infarction, support our conclusion that coronary disease risk factors, rather than patient sex, are more important determinants of type 2 myocardial infarction.^12^

Our study has several limitations. First, we recognize that our study population is limited to patients attending a secondary or tertiary care hospital with suspected acute coronary syndrome, and the prevalence of subsequent myocardial infarction is therefore higher than would be anticipated in the general population. As the incidence of type 2 myocardial infarction in the general population is low, we conducted this analysis in a higher-risk population to enable a fully adjusted multivariable analysis. Nevertheless, we believe that our population is relevant to address this question since all patients enrolled in our study were hospitalized for an acute illness, which is a prerequisite for type 2 myocardial infarction. Second, we did not have access to information on all cardiovascular risk factors, notably smoking status, and hypertension, nor did we have access to lipid profiles or blood pressure readings to evaluate the role of risk prediction tools such as the Framingham risk score. Further studies in a population undergoing screening for cardiovascular risk factors are needed to confirm our findings. Third, although all diagnoses in patients with troponin concentrations above the 99th centile were adjudicated according to the Universal Definition, there was moderate agreement between adjudicators (*K* = 0.7–0.8) and potential for diagnostic misclassification, especially in patients without serial troponin testing. Finally, our study was limited to a single healthcare system, and the prevalence of type 2 myocardial infarction is highly dependent on the approach to patient selection for troponin testing.^37^ As such, validation of our results in other healthcare settings is needed to confirm the generalizability of our findings.

## Conclusions

Traditional cardiovascular risk factors usually associated with type 1 myocardial infarction are also important predictors of type 2 myocardial infarction. Treatment and optimization of these risk factors may reduce the future risk of both type 1 and type 2 myocardial infarction.

## Supplementary material

Supplementary material is available at *European Heart Journal* online.

## Funding

This work was supported by several grant funding organizations. The High-STEACS trial was funded by a Special Project Grant from the British Heart Foundation (SP/12/10/29922). R.W. and A.B. are supported by Clinical Research Training Fellowship (MR/V007017/1 and MR/V007254/1) from the UK Medical Research Council and by a Research Excellence Award (RE/18/5/34216) from the British Heart Foundation. D.M.K. was supported by Health Data Research UK, which receives its funding from HDR UK Ltd (HDR-5012) funded by the UK Medical Research Council, Engineering and Physical Sciences Research Council, Economic and Social Research Council, Department of Health and Social Care (England), Chief Scientist Office of the Scottish Government Health and Social Care Directorates, Health and Social Care Research and Development Division (Welsh Government), Public Health Agency (Northern Ireland), British Heart Foundation, and the Wellcome Trust. Abbott Laboratories provided cardiac troponin assay reagents, calibrators, and controls without charge. N.L.M. is supported by the Butler BHF Senior Clinical Research Fellowship (FS/16/14/32023), a Programme Grant (RG/20/10/34966), and a Research Excellence Award (RE/18/5/34216) from the British Heart Foundation. A.R.C. receives support from a Starter Grant for Clinical Lecturers by the Academy of Medical Sciences (SGL021/1075).

**Conflict of interest:** N.L.M. has received honoraria from Abbott Diagnostics, Roche Diagnostics, Siemens Healthineers, and LumiraDx, and the University of Edinburgh has received research grants from Abbott Diagnostics and Siemens Healthineers. All other authors declared no conflict of interest.

## Data availability

The trial makes use of multiple routine electronic healthcare data sources that are linked, deidentified, and held in our national safe haven, which is accessible by approved individuals who have undertaken the necessary governance training. Summary data and the analysis code can be made available upon request to the corresponding author.

## Supplementary Material

ehab581_Supplementary_Data
