# Supplementary material for: Risk factors for type 1 and type 2 myocardial infarction
Source: Eur Heart J. 2021 Aug 25;43(2):127–35. doi: 10.1093/eurheartj/ehab581 (PMC8757580; doi:10.1093/eurheartj/ehab581)

# SUPPLEMENTARY APPENDIX

**Risk factors for type 1 and type 2 myocardial infarction**

Ryan Wereski MD *^a^*, Dorien M Kimenai PhD*^b^*, Anda Bularga MD *^a^*, Caelan Taggart MD*^a^*,

David J Lowe MD*^c^*, Nicholas L Mills MD PhD *^a,b^*, Andrew R Chapman MD PhD *^a^*

*on behalf of the High-STEACS Investigators*

*^a^ BHF Centre for Cardiovascular Science, University of Edinburgh, United Kingdom*

*^b^ Usher Institute, University of Edinburgh, United Kingdom*

*^c^ University of Glasgow, School of Medicine, Glasgow, United Kingdom*

**Correspondence and requests for reprints:**
Dr Andrew R Chapman

BHF Centre for Cardiovascular Science

Chancellors Building

Royal Infirmary of Edinburgh

Edinburgh EH16 4SA

United Kingdom

Tel: +44-131-242-6515

Fax: +44-131-242-6379

Email: [a.r.chapman@ed.ac.uk](mailto:a.r.chapman@ed.ac.uk)

Twitter: @chapdoc1

**Supplementary Appendix**

**Detailed description of diagnostic adjudication.**

All patients with hs-cTnI concentrations above the sex-specific 99th centile were classified according to the Third Universal Definition of Myocardial Infarction in use at the time of the trial. In this pre-specified secondary analysis, we updated this classification in accordance with the Fourth Universal Definition of Myocardial Infarction. The final diagnosis was adjudicated according to a pre-specified list (cardiac diagnoses: acute aortic dissection, acute heart failure, cardiomyopathy, chronic heart failure, hypertensive heart disease, myopericarditis, non-ST segment elevation myocardial infarction, ST-segment elevation myocardial infarction, recent myocardial infarction, tachyarrhythmia, Takotsubo cardiomyopathy or valvular heart disease; non-cardiac diagnoses: acute kidney injury, chronic kidney disease, chronic obstructive pulmonary disease, gastrointestinal bleed, pulmonary embolism, sepsis, or other). Two physicians independently reviewed all clinical information, blinded to study phase, with discordant diagnoses resolved by a third reviewer. Clinical information included the dates and times of presentation and final discharge, the initial emergency department assessment and final discharge letter as documented in the electronic care record, with summaries of all investigations undertaken during the index presentation including the electrocardiogram. The adjudication panel had access to raw clinical information including haemoglobin, creatinine, and high-sensitivity cardiac troponin I concentrations, and the reports from invasive coronary angiography. Type 1 myocardial infarction was defined as myocardial necrosis (any hs-cTnI concentration above the 99^th^ centile with a rise and/or fall in hs-cTnI concentration where serial testing was performed) in the context of a presentation with suspected acute coronary syndrome with symptoms or signs of myocardial ischemia on the electrocardiogram. Patients with symptoms or signs of myocardial ischemia and evidence of increased oxygen demand or decreased supply (for example, tachyarrhythmia, hypotension, or anaemia) secondary to an alternative pathology and myocardial necrosis were defined as type 2 myocardial infarction. The classification of type 2 myocardial infarction also includes patients with coronary vasospasm, embolism or spontaneous dissection without evidence of atherothrombosis related to coronary artery disease. Type 4a myocardial infarction was defined in patients with symptoms or signs of myocardial ischemia following percutaneous coronary intervention where hs-cTnI concentrations were 5-fold greater than the 99^th^ centile, or increased further if elevated prior to the procedure. Type 4b myocardial infarction was defined where myocardial ischemia and myocardial necrosis were associated with stent thrombosis documented at angiography. Myocardial injury was defined if hs-cTnI concentrations were above the 99^th^ centile in the absence of any clinical features of myocardial ischemia. All non-ischemic myocardial injury was classified as acute, unless a change of <20% was observed on serial testing or the final adjudicated diagnosis was chronic heart failure or chronic renal failure, where the classification was chronic myocardial injury.

**Table S1:** Adjudicated diagnosis at index presentation

|  | **All patients** | **No myocardial injury** | **Type 1 myocardial infarction** | **Type 2 myocardial infarction** | **Acute myocardial injury** | **Chronic myocardial injury** |
| --- | --- | --- | --- | --- | --- | --- |
| No. of participants | 48,282 | 37,922 | 4,981 | 1,121 | 1,676 | 1,287 |

**Table S2:** Baseline characteristics of patients with subsequent type 2 myocardial infarction, stratified by the primary cause of acute supply or demand imbalance

|  | **All patients** | **Primary mechanism of acute supply/demand imbalance** | | | | |
| --- | --- | --- | --- | --- | --- | --- |
|  |  | **Tachycardia** | **Hypoxemia** | **Hypotension** | **Anaemia** | **Hypertension** |
| No. of participants | 407 | 204 | 88 | 49 | 44 | 9 |
| Age (years), median (IQR) | 77 (69 – 83) | 76 (69 - 82) | 78 (68 - 83) | 79 (70 - 83) | 79 (72 - 84) | 76 (70 - 83) |
| Sex (Male) | 189 (46) | 90 (44) | 42 (48) | 24 (49) | 22 (50) | < 5 |
| Signs of myocardial ischaemia on 12-lead electrocardiogram | 157(39) | 76 (37) | 37 (42) | 20 (41) | 17 (39) | < 5 |
| Symptoms of myocardial ischaemia | 317 (78) | 173 (85) | 57 (65) | 33 (67) | 38 (86) | 9 (100) |
| *Medical history prior to index presentation* |  |  |  |  |  |  |
| Coronary artery disease | 236 (58) | 118 (58) | 55 (63) | 28 (57) | 25 (57) | < 5 |
| Cerebrovascular disease | 53 (13) | 23 (11) | 14 (16) | 9 (18) | 6 (14) | < 5 |
| Hyperlipidaemia | 273 (67) | 136 (67) | 60 (68) | 37 (76) | 30 (68) | < 5 |
| Diabetes mellitus | 85 (21) | 36 (18) | 21 (24) | 17 (34.7) | 9 (21) | < 5 |
| Creatinine concentration, median (IQR) | 92 (72 – 122) | 87 (68 - 108) | 98 (74 – 147) | 87 (75 – 109) | 85 (73 – 135) | 102 (76 – 119) |
| PCI | 63 (16) | 36 (18) | 9 (10) | 12 (25) | 5 (11) | 0 |
| CABG | 11 (3) | 5 (3) | < 5 | < 5 | < 5 | 0 |
| *Prior myocardial infarction or injury* |  |  |  |  |  |  |
| Type 1 MI | 83 (21) | 20 (10) | 13 (16) | < 5 | < 5 | 0 |
| Type 2 MI | 75 (19) | 11 (6) | 7 (9) | 5 (10) | < 5 | 0 |
| Acute myocardial injury | 38 (10) | 88 (45) | 31 (38) | 24 (50) | 14 (34) | < 5 |
| Chronic myocardial injury | 25 (7) | 32 (16) | 20 (25) | 11 (22.9) | 15 (37) | < 5 |
| No myocardial injury | 166 (43) | 44 (23) | 10 (12) | 5 (10) | 11 (27) | < 5 |
| *Medical therapies on subsequent admission* |  |  |  |  |  |  |
| ACE inhibitor/ ARB | 254 (62) | 136 (67) | 50 (57) | 34 (69) | 28 (64) | < 5 |
| Beta blocker | 262 (64) | 145 (71) | 50 (57) | 31 (63) | 27 (61) | < 5 |
| Aspirin | 265 (65) | 134 (66) | 54 (61) | 31 (63) | 33 (75) | 5 (56) |
| PY12 inhibitor | 135 (32) | 59 (29) | 32 (36) | 18 (37) | 19 (43) | < 5 |
| Oral anticoagulant | 103 (25) | 68 (33) | 22 (25) | 7 (14) | 5 (11) | < 5 |
| Spironolactone | 42 (10) | 25 (12) | 11 (13) | < 5 | < 5 | 0 |
| Lipid lowering therapy | 297 (73) | 148 (73) | 63 (72) | 38 (78) | 36 (82) | < 5 |

**Table S3.** Characteristics of patients with recurrent type 1 or type 2 myocardial infarction

|  | **Type 1 myocardial infarction on index and subsequent admission** | **Type 2 myocardial infarction on index and subsequent admission** | **P value** |
| --- | --- | --- | --- |
| No. of participants | 409 | 75 |  |
| Age (years), median (IQR) | 74 [63 – 83] | 77 [70.5 – 83] | 0.04 |
| Sex (Male) | 218 (53) | 28 (37) | 0.016 |
| *Medical history prior to index presentation* |  |  |  |
| Coronary artery disease | 206 (50) | 42 (56) | 0.440 |
| Cerebrovascular disease | 49 (12) | 8 (10.7) | 0.897 |
| Hyperlipidaemia | 252 (62) | 45 (60) | 0.893 |
| Diabetes mellitus | 92 (23) | 9 (12) | 0.057 |
| Abnormal renal function | 170 (43) | 31 (43) | 1.0 |
| Creatinine concentration, median (IQR) | 89 [73 – 116] | 85 [69 – 107] | 1.0 |
| PCI | 64 (16) | 12 (16) | 1.0 |
| CABG | 19 (5) | < 5 | 1.0 |
| *Treatment on index admission* |  |  |  |
| Angiography | 200 (49) | 12 (16) |  |
| PCI | 123 (30) | <5 |  |
| CABG | 5 (1) | 0 |  |
| *Medical therapies on subsequent admission* |  |  |  |
| ACE inhibitor/ ARB | 301 (74) | 38 (51) | <0.001 |
| Beta blocker | 286 (70) | 51 (68) | 0.844 |
| Aspirin | 329 (80) | 45 (60) | <0.001 |
| PY12 inhibitor | 299 (73) | 23 (31) | <0.001 |
| Oral anticoagulant | 39 (10) | 31 (41) | <0.001 |
| Spironolactone | 35 (9) | 7 (9) | 1.0 |
| Lipid lowering therapy | 340 (83) | 55 (73) | 0.064 |

**Table S4.** Data sources used to capture population demographics and diagnoses

|  | **Data source(s)** | | |
| --- | --- | --- | --- |
| **Variable** | **ICD-10** | **OPCS-4** | **Other** |
| Age | - | - | Electronic patient record |
| Sex | - | - | Electronic patient record |
| Coronary artery disease | I20-I25 | - | - |
| Cerebrovascular disease | I60-I68 | - | - |
| Hyperlipidaemia |  |  | National prescribing register |
| Diabetes mellitus | E10-E14 | - | Scottish national diabetes register  (SCI-Diabetes) |
| Creatinine concentration |  |  | Electronic patient record |
| Previous PCI | - | K49,K50,K75 | TOMCAT and CATHI angiography databases |
| Previous CABG | - | K40-48 | - |
| Prior type 1 myocardial infarction | - | - | Adjudication of index presentation |
| Prior type 2 myocardial infarction | - | - | Adjudication of index presentation |

**Figure S1.** Aetiology of supply or demand imbalance in patients with subsequent type 2 myocardial infarction events


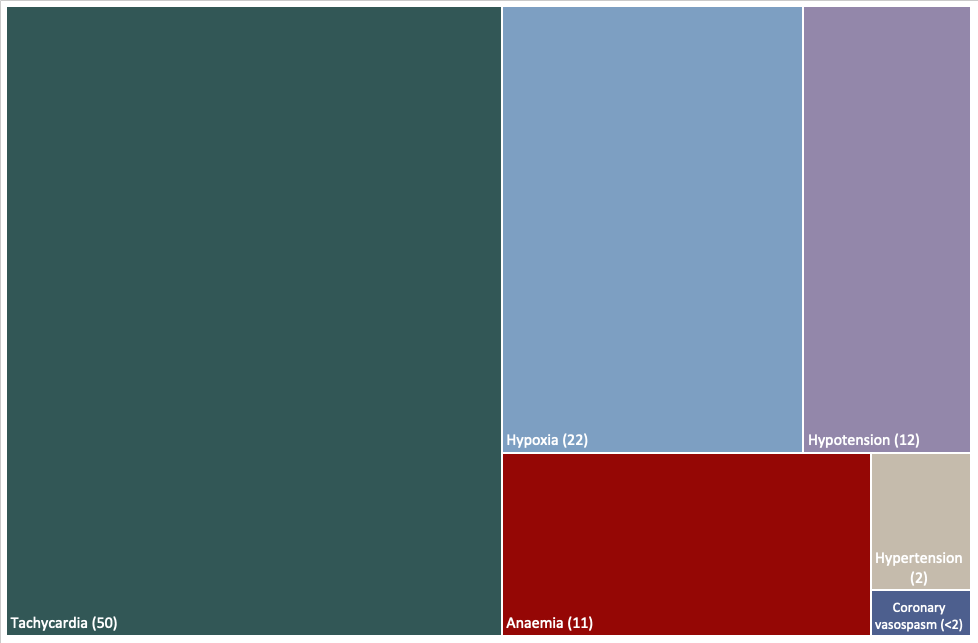

Supplement: ehab581_Supplementary_Data [file ehab581_supplementary_data.docx]
